# Delayed Resolution of Acute Inflammation in Ulcerative Colitis Is Associated with Elevated Cytokine Release Downstream of TLR4

**DOI:** 10.1371/journal.pone.0009891

**Published:** 2010-03-26

**Authors:** Farooq Z. Rahman, Andrew M. Smith, Bu'Hussain Hayee, Daniel J. B. Marks, Stuart L. Bloom, Anthony W. Segal

**Affiliations:** 1 Departments of Medicine, University College London, London, United Kingdom; 2 Department of Gastroenterology, University College London Hospital, London, United Kingdom; Charité-Universitätsmedizin Berlin, Germany

## Abstract

**Background:**

Ulcerative colitis (UC) is widely viewed as a leukocyte-mediated disorder. Although strong evidence implicates an exuberant response to microbial components in its pathogenesis, no intrinsic immune defect has been identified and the underlying pathogenic mechanisms remain obscure.

**Methodology/Principal Findings:**

The acute immune response to bacterial injection was determined in UC patients with quiescent disease and directly compared to healthy control subjects. Monocyte-derived macrophages were used to investigate bacterial recognition mechanisms *in vitro*. An exuberant and protracted acute inflammatory response to bacteria was evident in patients with UC, which coincides with increased systemic levels of CXCL10. Macrophages stimulated with bacteria and Toll-like receptor (TLR) ligands revealed a specific defect in the TLR4 response in UC. The defect resulted in the over-expression of a number of pro-inflammatory molecules under transcriptional control of the adaptor TIR-domain containing adaptor inducing interferon-β (TRIF).

**Conclusion:**

These findings highlight a dysregulated innate immune response with over-expression of molecules associated with leukocyte recruitment and activation that may eventuate in the hallmark chronic immune-mediated inflammation of UC.

## Introduction

Ulcerative colitis (UC) is a chronic inflammatory bowel disease (IBD) characterised by intermittent florid, continuous superficial colonic inflammation. It has an incidence of 1 in 1,000 and is associated with considerable morbidity including an increased long-term risk of colorectal cancer. UC is most commonly confined to the rectum or left colon, but involves the whole colon in 20% of cases. A small proportion of patients (5%) have rectal sparing and/or backwash ileitis. Aminosalicylates (5-ASA), corticosteroids and immunomodulators thought to intervene at various points along the immune and inflammatory cascade are the mainstay of medical therapy. Colectomy for severe or recalcitrant disease is required in 20% of patients[1].

It is currently believed that an aberrant innate immune response to commensal colonic microbiota in genetically susceptible individuals is a prerequisite for excessive activation of adaptive immunity within the lamina propria[2]. Although this concept is supported by extensive evidence from human and animal studies, neither an intrinsic immune defect nor the underlying mechanism(s) have been identified to date. The pathogenesis of UC is clearly complex and likely to involve multiple genetic, environmental and immunological factors[3]. Furthermore, recent genome wide association (GWA) studies in UC[4], [5], [6] have provided few direct clues as to the aetiology. Macrophages are crucially important in orchestrating innate and adaptive immune responses to microbes via pattern recognition receptors (TLRs and NOD-like receptors). These sentinels of the cellular innate immune system recognise pathogen associated microbial patterns and mediate diverse immune responses through interconnected, but subtly distinct, signalling pathways[7]. Colonic microbiota provide the innate immune system, and specifically TLRs, with the complex challenge of balancing tolerance and immunological responsiveness to infection. A dysregulated TLR-mediated innate immune response may be central to the pathogenesis of UC and produce the immune cell-mediated inflammation characteristic of this disease.

In this study, we examined the acute inflammatory response to bacterial challenge in UC. Using an *in vivo* model of acute inflammation, we demonstrate dysregulated innate immunity in UC with an exuberant, protracted acute inflammatory response to subcutaneously injected heat killed *E.coli* (HkEc), with a concurrent elevation in CXCL10 in the circulation. To elucidate the underlying cellular mechanism we then examined cytokine secretion and gene expression in primary monocyte-derived macrophages stimulated with HkEc and individual TLR ligands. These *in vitro* studies identify a specific defect relating to TLR4, which results in over-expression of a number of pro-inflammatory molecules associated with leukocyte recruitment and activation.

## Materials And Methods

### Ethics Statement

These studies were approved by the Joint UCL/UCLH Committee for the Ethics of Human Research (project number 04/0324 and 05/Q0505/115). Written informed consent was obtained from all volunteers.

### Subjects

Adult patients with definitive diagnoses of UC confirmed using standard diagnostic criteria, who met inclusion criteria, were recruited from the Gastroenterology outpatient clinic at University College London Hospitals NHS Foundation Trust (UCLH). All patients had quiescent disease (Mayo score <3 and serum inflammatory markers within normal limits) and were on either no medication or mesalazine alone (≤2.4 g/day) at a stable dose for at least 3 months prior to recruitment. No patient had received corticosteroid or biological therapy within three months of inclusion. Healthy controls were approximately matched for age, sex and smoking history. No subject was studied more than once in any of the different sets of experiments.

### Subcutaneous injection of heat killed E. coli

For bacterial injection experiments, a fully antibiotic-sensitive clinical isolate of *E. coli* was grown overnight in minimal citrate medium supplemented with 1.25 g/L yeast extract (Oxoid), then killed by heating to 80°C for 30 minutes. Bacteria were washed twice in sterile PBS, aliquoted, centrifuged and the pellets were snap-frozen and stored at −70°C. Sterility was confirmed by culture. Bacteria were resuspended in injection grade normal saline. Initial dose response was carried out using 2.5×10^8^, 6×10^7^, 3×10^7^, 1.5×10^7^ and 7.5×10^6^ *E. coli* per 100 µl. Four HC subjects were injected subcutaneously into the volar aspect of each forearm with four of the doses. From the dose response results all subsequent studies used 3×10^7^ per 100 µl. Blood was collected before and 24, 48 and 72 h after injection, for full blood counts and measurements of C-reactive protein (CRP) and serum cytokines (see below). Blood flow was measured at the injection sites by laser Doppler imaging, and the mean reading of the two forearms taken (MoorLDI2; Moor Instruments, Axminster, UK).

### Serum cytokine measurements

The expression profile of a panel of cytokines in serum samples collected at 24, 48 and 72 h post inoculation were measured using the Beadlyte Bio-Plex™ human cytokine assay (Bio-Rad), according to the manufacturer's instructions.

### Macrophage isolation and culture and stimulation

Peripheral venous blood was collected from subjects into syringes containing 5 U/ml heparin. Mononuclear cells were isolated by differential centrifugation (900 g, 30 minutes, 20°C) over Lymphoprep and washed twice with sterile phosphate-buffered saline (PBS; GIBCO, Paisley, UK) at 300 g (5 minutes, 20°C). Cells were resuspended in 10 ml RPMI-1640 medium (Invitrogen) supplemented with 100 U/ml of penicillin (GIBCO), 100 µg/ml streptomycin (GIBCO) and 20 mM HEPES pH 7.4 (Sigma-Aldrich), and plated at a density of approximately 5×10^6^ cells/ml in 8 cm^2^ NunclonTM Surface tissue culture dishes (Nunc, Roskilde, Denmark) at 37°C, 5% CO_2_. After 2 h, non-adherent cells were discarded and 10 ml of fresh RPMI supplemented with 10% foetal bovine serum (FBS; Sigma) added to each tissue culture dish. Cells were then cultured for 5 days at 37°C, 5% CO_2_, with the addition of a further 10 mls of fresh 10% FBS/RPMI after 24 h.

Adherent cells were scraped on day 5 and re-plated in 96-well culture plates at equal densities (10^5^/well) in X-Vivo-15 medium (Cambrex, Walkersville, MD, USA). These primary monocyte-derived macrophages were incubated overnight at 37°C, 5% CO_2_ to adhere, and then stimulated for up to 24 h with either 2.5×10^5^ HkEc, (preparation method same as above), Pam_2_CSK_4_ (2 µg/ml), Pam_3_CSK_4_ (2 µg/ml), smooth LPS (sLPS, 200 ng/ml), rough LPS (rLPS, 200 ng/ml) Poly I:C (125 µg/ml) or flagellin (500 ng/ml) (Alexis Biochemicals, San Diego, US).

### Cytokine secretion assays

Macrophage supernatants were collected after 24 h stimulation of primary monocyte-derived macrophages with HkEc, Pam_2_-CSK_4_, Pam_3_-CSK_4_, sLPS, rLPS, poly I:C or flagellin as described previously. The expression profile of a panel of cytokines in macrophage supernatants was measured using the Beadlyte Bio-Plex™ human cytokine assay (IL-1Ra, IL-4, IL-5, IL-6, IL-10, IL-12, IL-13, IL-15, IL-17, G-CSF, GM-CSF, IFN-γ, CXCL10 and MCP-1) (Bio-Rad), according to the manufacturer's instructions. IL-8 secretion was measured using commercially available sandwich ELISA Development kits (R&D Systems, Abingdon, UK).

TNF-α release was measured using a cytotoxicity bioassay (obtained from Prof. B. Beutler, The Scripps Institute, CA, USA). Murine L929 fibroblast cells were grown in DMEM (GIBCO), supplemented with 10% FBS (Sigma), 100 U/ml of penicillin (GIBCO) and 100 µg/ml streptomycin (GIBCO), at 37°C, 5% CO_2_. A confluent monolayer of murine L929 fibroblasts was trypsinised and resuspended to 4×10^5^ cells/ml in DMEM. L929 cells were seeded into 96-well flat bottomed tissue culture plates (4×10^4^ cells/well), and incubated overnight at 37°C, 5% CO_2_. After overnight culture, the medium was discarded, replaced by 50 µl DMEM containing cyclohexamide (0.04 mg/ml) and incubated for 20 minutes at 37°C, 5% CO_2_. 50 µl of cell-free supernatant (diluted 1∶50 in DMEM), collected from primary macrophages as already described, were added to individual wells. Serially diluted recombinant human TNF-α (100-0 pg/ml) was used to determine the standard curve for the assay.

IFN-β release was determined using an L-929 cell line (4×10^5^ cells/well) transfected with an IFN-sensitive luciferase construct (obtained from Prof B. Beutler)[8]. Results expressed as relative luminescence per ml (rl/ml).

Cytokine release into culture supernatants was normalized for the numbers of viable cells in each well, ascertained with the MTT (3-[4,5-dimethylthiazol-2-yl]-2,5-diphenyl tetrozolium bromide, tetrazolium salt) assay (Boehringer Ingelheim, Berkshire, UK). 20 µl of 2.5 ng/ml MTT was added to each well and incubated for 4 h at 37°C, 5% CO_2_. Supernatants were carefully discarded and 100 µl/well of lysis solution (90% isopropanol, 0.5% sodium dodecyl sulphate, 0.04 N HCl, 10% H_2_0) added to each well for 1 h at room temperature. The absorbance was read at 570 nm using a microplate reader (FLUOstar-omega, BMG labtech, UK).

### RNA purification

Total RNA was prepared from monocyte-derived macrophages after 4 h stimulation with HkEc, using the RNeasy Mini Kit with RNase–free DNase treatment (Qiagen GmbH, Hilden, Germany). Optical density readings were determined for OD_260_/OD_280_ and OD_260_/OD_230_ using a NanoDrop ND-1000 spectrophotometer (Fisher Scientific, Loughborough, UK) to assess protein and solvent contamination respectively.

### Toll-like receptor pathway OligoGEarray chips

Macrophage total RNA was prepared as previously described, both unstimulated and following incubation with HkEc. Total RNA (1 µg) was processed to produce biotinylated cRNA targets, using the TrueLabeling-AMP™ 2.0 Kit (Superarray Bioscience, MD, USA), according to the manufacturer's instructions. Double-stranded cDNA was synthesized by reverse transcription of twice-purified mRNA using Truelabeling primers and cDNA Synthesis Master Mix (Superarray Bioscience, MD, USA). The cDNA was used as a template to generate biotin-labelled *in vitro* transcription products using an Amplification Master Mix (Superarray Bioscience, MD, USA), and biotin-11-UTP (PerkinElmer, MD, USA). Biotinylated cRNA products were purified using a Superarray ArrayGrade cRNA Cleanup Kit and quantified using a Nanodrop® spectrophotometer as previously described. Biotinylated cRNA (2 µg) was hybridzed to Oligo GEArrays® following standard Superarray protocol. Microarrays were analyzed using the manufacturer's web-based integrated data analysis software (GEArray Expression Analysis Suite).

### Statistical analysis

All data are presented as mean ± SEM. Statistical significance between groups was evaluated using an unpaired two-tailed Student's t-test. Mean differences were considered significant when p<0.05.

## Results

### E. coli challenge in vivo

Previously, one UC patient we studied developed a severe reaction to injected bacteria with persistent local inflammation leading to ulceration at both inoculation sites[9]. This reaction occurred after an injection of 10^9^ HkEc, so further studies were only permissible at the lowest dose capable of eliciting a detectable response in healthy controls (HC), this corresponded to 3×10^7^ organisms per inoculation (Figure S1). The subsequent local and systemic acute inflammatory responses in 21 UC patients and 15 HC subjects (Table S1) were determined.

### Local blood flow

**Figure 1:**
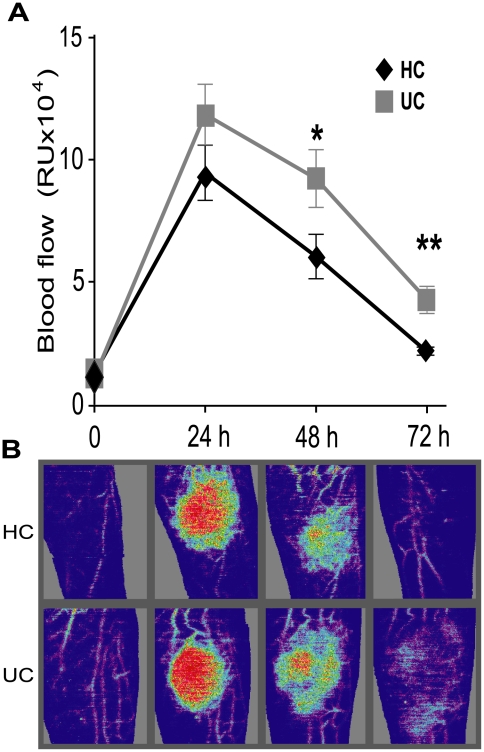
Local inflammation was protracted in UC patients after subcutaneous injection of HkEc. (**A**) Blood flow at the injection site was elevated and prolonged in patients with UC (n = 21) at 48 (p = 0.04) and 72 h (p = 0.002) compared to HC (n = 15). (**B**) Laser Doppler images showing elevated and prolonged local blood flow in a UC patient after HkEc inoculation (Red represents high and purple low blood flow).

The magnitude of the acute inflammatory response was quantified by measuring elevation in local blood flow following bacterial challenge. This was maximal in both groups at 24 h, returning to baseline by 72 h in HC, with no difference in peak response between patients and controls (Figure 1). UC patients demonstrated a prolonged response with significantly elevated blood flow at 48 (p = 0.04) and 72 (p = 0.002) h. Results were not significantly different in UC patients with intact colons (n = 16) compared to those that had undergone colectomy (n = 5) (Figure S2). These findings provide strong evidence for an abnormal systemic inflammatory response to bacteria in UC patients, mediated by circulating marrow factors rather than colonic tissue.

### Systemic acute phase inflammatory response to E. coli challenge

**Figure 2:**
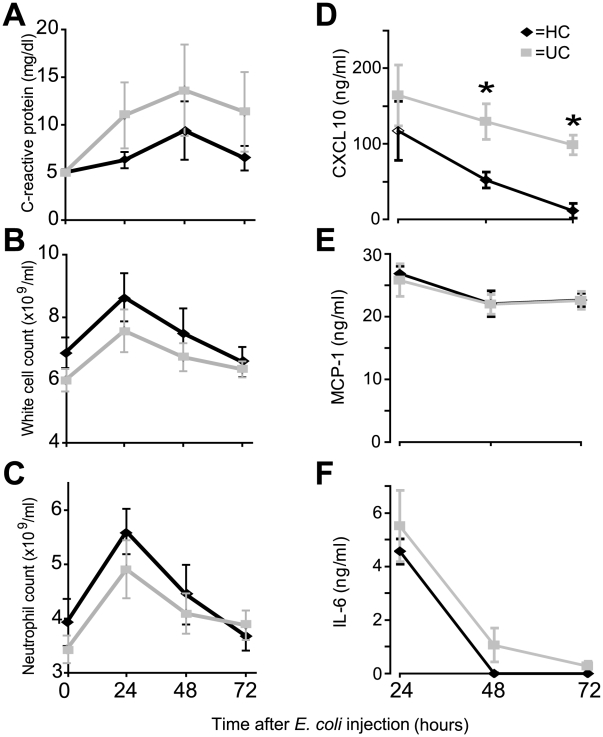
Systemic CXCL10 levels were elevated in UC patients. Effects of subcutaneous injection of HkEc on, (**A**) C-reactive protein (all subjects had baseline CRP levels of <5 mg/dl), (**B**) white cell count, (**C**) circulating neutrophil numbers, (**D**) CXCL10, (**E**) MCP and (**F**) -1 IL-6. TNF-α, IFN-γ, IL-8, IL-10, IL-13, IL-17 and MIP-1α were undetectable in serum samples at all time-points. Results are expressed as a mean ± SEM (*p<0.05).

The systemic acute phase response was similar in the two groups: CRP levels were elevated in both and maximal at 48 h (Figure 2A). White cell and neutrophil counts peaked at 24 h before returning to baseline after 72 h (Figure 2B, C) in both groups.

### Serum cytokine/chemokine changes after E. coli challenge

We next measured systemic cytokine/chemokine levels in the serum (Figure 2 D–F). Circulating CXCL10 levels were significantly greater at 48 (p = 0.02) and 72 h (p = 0.03) after HkEc inoculation in UC, which coincided with elevated blood flow. IL-6 and MCP-1 levels were not significantly different between the two groups over the 72 h period. TNF, IFN-γ, IL-8, IL-10, IL-13, IL-17 and MIP-1α were undetectable in serum samples at all time-points.

### Macrophage cytokine release after E. coli stimulation

**Figure 3:**
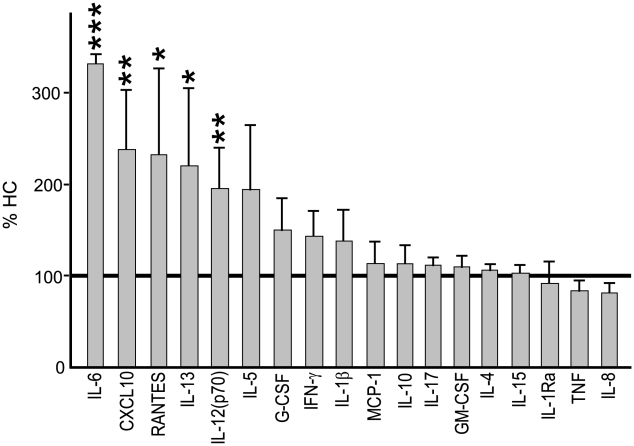
Pro-inflammatory cytokine secretion by UC macrophages (n = 11) in response to HkEc. Results are expressed as a percentage (mean ± SEM) of that secreted by HC cells (absolute values for HC shown in Table S1). (*p<0.05, **p<0.01 and ***p<0.001).

Our results show a prolonged local inflammatory response with elevated systemic CXCL10 levels in UC patients following bacterial stimulation. To investigate these phenomena in more detail we studied monocyte-derived macrophages from UC (n = 17) and HC (n = 11) subjects. Cells were exposed to HkEc for 24 h and the cytokine/chemokine profile determined (Figure 3). Release of CXCL10, RANTES, IL-12_p70_, IL-13 and IL-6 was significantly elevated in UC. In contrast, no significant differences were observed in the secretion levels of TNF, GM-CSF, G-CSF, IFN-γ, IL-8, MCP-1, IL-4, IL-5, IL-15, IL-17, IL-10 and IL-1ra between the two groups. These results provide evidence that macrophages from UC patients release significantly higher amounts of a selective group of chemokines/cytokines following bacterial stimulation.

### Macrophage cytokine/chemokine secretion following TLR stimulation

**Figure 4:**
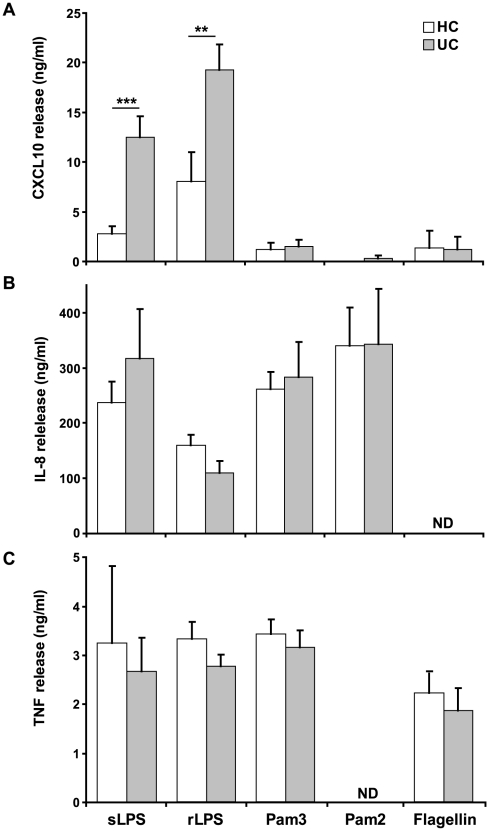
Macrophages from UC patients demonstrate defective cytokine release downstream of TLR4. Macrophages from UC (n = 12) and HC (n = 8) were stimulated for 24 h with sLPS (TLR4/CD14), rLPS (TLR4), Pam_3_CSK_4_ (TLR2/1), Pam_2_CSK_4_ (TLR2/6) and flagellin (TLR5). Secreted levels of (**A**) CXCL10, (**B**) IL-8 and (**C**) TNF-α were measured. Results are expressed as a mean ± SEM (**p<0.01, ***p<0.001) (ND  =  not determined).

Whole bacteria are capable of activating a number of macrophage receptors including TLR and NOD-like receptors. It is possible that the defective cytokine release is due to an abnormality in the signalling pathways associated with one or more of these receptors. Highly purified bacterial-derived TLR ligands were used to investigate the nature of the defective immune response to bacteria, these included; rough-LPS (rLPS) (TLR4), smooth-LPS (sLPS) (TLR4/CD14), Pam_3_-CSK_4_ (TLR2/1) Pam_2_-CSK_4_ (TLR2/6) and flagellin (TLR5). Macrophages from UC patients released significantly elevated levels of CXCL10 following sLPS and rLPS (Figure 4A). Exposure to Pam_3_-CSK_4_, Pam_2_-CSK_4_ and flagellin resulted in low CXCL10 secretion with equivalent levels in both UC and HC (Figure 4A). The fact that both sLPS and rLPS resulted in elevated CXCL10 suggests that the defective TLR4 response is not CD14-dependent. In contrast to CXCL10, release of IL-8 and TNF-α were not significantly different in the two groups following stimulation with any of the TLR ligands (Figure 4B and C). This result suggests that defective macrophage cytokine secretion in UC results from an abnormality of TLR4, and not TLR 1, 2, 5 or 6, signalling.

### Elevated Type I interferon release downstream of TLR4

**Figure 5:**
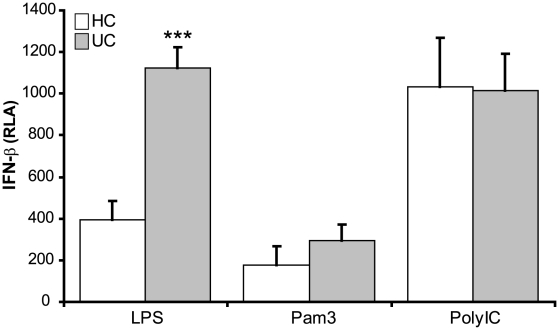
TLR4 activation results in an elevation in TRIF signaling and subsequent IFN-β release by macrophage from UC patients. Macrophages from UC (n = 11) and HC (n = 7) were stimulated for 6 h with rLPS (TLR4), Pam_3_CSK_4_ (TLR2/1) or Poly I:C (TLR3) and the levels of IFN-β release in determined. Results are expressed as relative luciferase activity (RLA) (mean ± SEM, ***p<0.001).

TLR4, unlike other TLRs, is unique in its ability to activate MyD88-dependent and TRIF-dependent signalling pathways[10]. TRIF activates the transcription factor IRF3, which induces genes that contain interferon-sensitive response elements (ISRE). Genes which are dependent on IRF3 include the type I interferons (IFN-α and -β) which are release within hours after TLR stimulation[11]. In order to determine whether the abnormal TLR4 response in UC relates to a defective TRIF pathway, macrophages were stimulated for 6 h with LPS and Pam_3_-CSK_4_ and the level of IFN-β determined using an ISRE-luciferase reporter assay (Figure 5). Induction of the MyD88-dependent pathway via TLR2/1 resulted in minimal IFN-β release in both UC and HC subjects. In contrast, simultaneous activation of both TRIF- and MyD88-dependent pathways through TLR4 resulted in the induction of IFN-β in both HC and UC. Macrophages from UC subjects released significantly more IFN-β than HC (p = 0.00006). To ascertain whether TRIF-dependent signalling is generally abnormal in macrophages from UC subjects, we stimulated cells with poly I:C, a TLR3 ligand (Figure 5). Activation of TLR3, which results in selective activation of the TRIF-dependent pathway resulted in release of equivalent levels of IFN-β in HC and UC, suggesting a TLR4-specific macrophage defect in UC.

### Macrophage gene expression after heat killed E.coli stimulation

**Figure 6:**
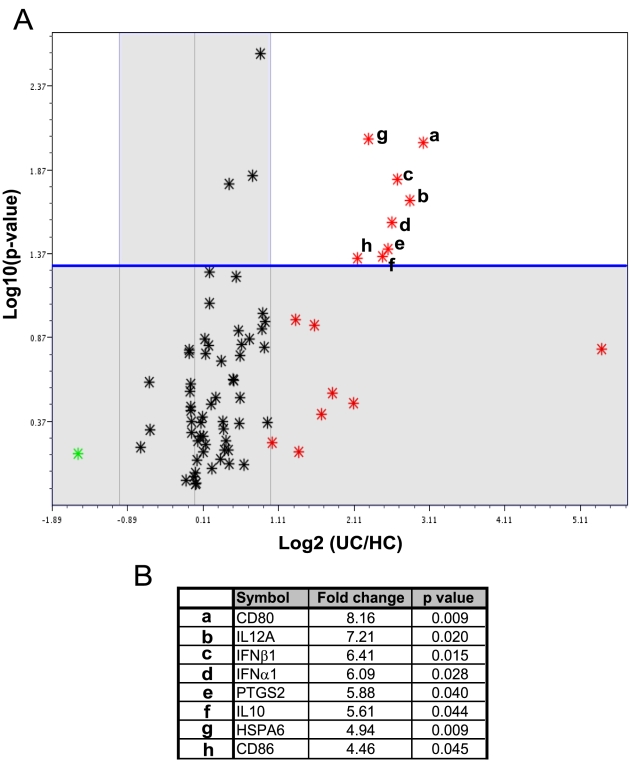
TLR-specific gene transcription profiles reveal increased expression of TRIF-responsive genes in macrophages from UC patients after stimulation with HkEc. Macrophages from UC (n = 8) and HC (n = 8) were stimulated for 4 h with HkEc followed by gene array analysis. (**A**) Volcano plot showing the alteration in expression levels between UC and HC after HkEc stimulation. Only eight genes (p<0.05 (blue line) and fold change >2) demonstrated significantly different expression levels after stimulation (labeled a–h). (**B**) Identification of the abnormally expressed genes with the corresponding fold change and p-value.

In order to discover if the hyperactive TLR4-TRIF dependent pathway was still evident when macrophages were exposed to whole bacteria transcription profiles following HkEc stimulation for 4 h were studies (Figure 6). We identified significant (p<0.05) over-expression (fold change >2) of a relatively small group of TLR-related genes (9 out of 123) in UC (n = 8) following stimulation with HkEc compared to HC (n = 8) (Figure 6). The abnormal gene list included the cytokines IFN-α, IFN-β, IL-12(p35), IL-10, MCP-1, heat shock protein HSPA6, cyclooxygenase 2 (COX2/PTGS2), as well as the T cell co-stimulatory molecules CD80 and CD86. The elevation in type I IFN expression provides evidence that exposure to whole bacterial results in over-activation of the TLR4-TRIF-dependent pathway. Interestingly, all of the over-expressed genes have previously been shown to be induced by TLR4-TRIF-dependent signaling[12], [13], [11], [14], [15].

## Discussion

In this study we have identified a dysfunctional systemic innate immune response to bacterial challenge in patients with UC. The abnormal acute inflammatory response in these patients manifests as an exuberant and protracted inflammatory response following bacterial challenge. Detailed *in vitro* studies identified TLR4 as the defective bacterial receptor on macrophages, more specifically, related to regulation of the TRIF-dependent signaling pathway. Defective signaling resulted in the elevation in type I IFNs as well as antigen presentation and co-stimulatory genes which have been associated with leukocyte recruitment and activation. This abnormal immune response to bacteria may play a role in the induction of the chronic inflammatory state characteristic of UC.

Although leukocyte-mediated chronic inflammation is a hallmark of UC, no intrinsic defect has been identified in leukocytes from these patients. GWA studies have recently uncovered susceptibility loci which may play a role in the pathogenesis of UC[4], [5], [6]. A high proportion of these contain genes involved in T cell-mediated immunity[16]. Although this further strengthens the concept of an aberrant immune response in UC, the pathogenic mechanism remains unclear. It is becoming increasingly apparent that UC and Crohn's disease (the other major inflammatory bowel disorder) arise from distinctly different immune defects. We have previously demonstrated an attenuated innate immune response in Crohn's which results from diminished cytokine secretion and the retention of bacteria in the tissue[17], [18]. This contrasts entirely with UC, where acute inflammation is prolonged after bacterial stimulation. For this reason, the exuberant response in UC patients is unlikely to be secondary to prior bacterial exposure or chronic bowel inflammation as the same argument should apply in Crohn's disease, in which bowel ulceration and exposure to coliform bacteria is similar[17].

Raised levels of numerous cytokines and chemokines have previously been reported in the serum and biopsies of patients with active UC[19], [20], [21], [22]. Of the serum cytokines measured following HkEc injection, only CXCL10 was shown to be upregulated systemically in UC subjects (the majority of cytokines were below detectable limits). Local blood flow and CXCL10 were both significantly elevated at 48 and 72 h post HkEc injection. CXCL10 is a potent chemoattractant secreted mainly by monocytes and macrophages within inflamed bowel[22]. CXCR3, the only known receptor for CXCL10, is present on lymphoid, epithelial and endothelial cells. CXCR3^+^ T cells are activated and recruited into inflammatory sites by CXCL10 and have been previously associated with colitis in mice[23], [24], [25]. Raised CXCL10 levels have been described both locally in colonic mucosal biopsies[22], and systemically in serum of patients with UC[26]. Interestingly, *ex vivo* studies on colonic biopsies identified macrophages rather than epithelial cells as the major source of CXCL10[22]. Our results support the notion that macrophages exposed to gram-negative bacteria alone have the potential to cause an exuberant and protracted inflammatory response and raised CXCL10 levels in UC patients.

Mouse models of colitis provide further support for a pathogenic role of CXCL10 in UC. CXCL10 and its receptor CXCR3 have been shown to be upregulated in IL-10 knockout (IL-10^−/−^) mice and those with dextran sulphate sodium (DSS)-induced colitis. Blockade of the CXCL10-CXCR3 axis with monoclonal anti-CXCL10 antibodies ameliorates colitis in IL-10^−/−^ mice by decreasing infiltrating T cells[25], and confers protection from DSS-induced colitis[23]. These findings have resulted in the development of novel therapeutics which target either CXCL10 or its receptor CXCR3[24]. A fully humanised anti-CXCL10 monoclonal antibody is currently undergoing phase 2 clinical trials in active UC.

Our results clearly show that the aberrant macrophage response to microbial challenge in UC relates specifically to TLR4 pathway dysregulation. In addition to CXCL10, UC macrophages also secrete elevated levels of a number of chemokines/cytokines following both HkEc and LPS stimulation. This contrasts with normal cytokine secretion following stimulation with other TLR ligands supporting a TLR4 specific defect in UC. TLR4 is unique as the only TLR capable of activating both MyD88- and TRIF-dependent signaling pathways[14]. The signalling defect in UC seems to lie specifically downstream of TLR4 TRIF-dependent pathway, with normal MyD88-dependent pathway gene expression. Stimulation of the TRIF-dependent pathway downstream of TLR3 results in normal cytokine production confirms a TLR4 TRIF-pathway defect in UC. Increased secretion of type I IFN could explain the elevation in CXCL10, RANTES, CD80 and CD86 expression due to IFN receptor (IFNR)-mediated auto-/paracrine feedback mechanisms[27], [28], [11], [14]. IL-6 and IL-12 expression has been shown to be controlled by a TLR4-TRIF-dependent/IFNR-independent signal[14]. Collectively these results suggest UC macrophages possess a defect in the TLR4-TRIF-dependent pathway that may be amplified by IFNR signalling.

TRIF-dependent genes over-expressed in UC macrophages following HkEc stimulation, include the Type I IFNs (IFNα1 and IFNβ1) and the T cell co-stimulatory molecules CD80 and CD86[8]. These molecules have been previously linked to bowel inflammation. Interestingly, *de novo* cases of bowel inflammation resembling UC have been described following recombinant Type I IFN treatment of chronic hepatitis and multiple sclerosis[29], [30], [31]. Type I IFN therapy have previously been trialed in UC yielding equivocal results[32], [33]. In addition, a direct pathological role for CD80 and CD86 in UC has been postulated[34], and blockade of CD80 suppresses colonic inflammation in a murine colitis model[35]. It is possible that dysregulated TLR4-TRIF signaling in UC distorts the immune response to bacteria with pathological consequences. Although the precise underlying mechanisms controlling the TRIF-dependent pathway downstream of TLR4 are still unclear, microbial and environmental stress have been shown to play a major role[36], [37]. It is therefore probable that directly targeting molecules associated with the TRIF-pathway may prove advantageous in the treatment of UC.

In conclusion, prolonged inflammation and elevated cytokine release are associated with an abnormal response to bacterial exposure downstream of TLR4 signalling. Macrophages from patients with UC have an aberrant TLR4-mediated LPS response that relates to dysregulated TRIF-dependent gene activation, resulting in the over-expression of molecules associated with leukocyte recruitment and activation. The abnormalities identified might eventuate in the chronic T cell-mediated inflammation that is a hallmark of UC. Future challenges include further characterization of the abnormal TLR4 TRIF-dependent signaling pathway in UC and identification of the precise molecular defects in UC, paving the way for novel therapeutics for this debilitating condition.

## Supporting Information

Figure S1 Dose response of local blood flow in HC subjects to a subcutaneous injection of E. coli.(A) Laser Doppler images of blood flow levels 24 h post injection. The dose, indicated above each panel, represents the total amount of bacteria injected. (B) Change in blood flow at the inoculation site over a 72 h period.
(2.02 MB EPS)

Figure S2 Comparing the effects of subcutaneous injection of E. coli on local inflammation in UC patients who have undergone colectomy surgery (n = 5) or have an intact colon (n = 16).
(1.73 MB EPS)

Table S1 HkEc injection study subject demographics;(A) Cumulative data, (B) HC, (C) UC. (*panproctocolectomy + ileoanal pouch).
(1.17 MB EPS)
